# Monitoring Urban Beach Quality on a Summer Day: Determination of the Origin of Fecal Indicator Bacteria and Antimicrobial Resistance at Prophète Beach, Marseille (France)

**DOI:** 10.3389/fmicb.2021.710346

**Published:** 2021-08-25

**Authors:** Mylène Toubiana, Christian Salles, Marie-George Tournoud, Patricia Licznar-Fajardo, Isabelle Zorgniotti, Marie-Laure Trémélo, Estelle Jumas-Bilak, Samuel Robert, Patrick Monfort

**Affiliations:** ^1^HydroSciences Montpellier, UMR 5151 Université de Montpellier, CNRS, IRD, Montpellier, France; ^2^Département d’Hygiène Hospitalière, CHU Montpellier, Montpellier, France; ^3^ESPACE, UMR 7300 Aix Marseille Université, Avignon Université, Université Côte d’Azur, CNRS, Aix-en-Provence, France

**Keywords:** fecal indicator bacteria, microbial source tracking, antimicrobial resistance, beach water, sand, attendance

## Abstract

A highly frequented beach in Marseille, France, was monitored on an hourly basis during a summer day in July 2018, to determine possible water and sand fecal pollution, in parallel with influx of beach users from 8 a.m. to 8 p.m. Fecal indicator bacteria were enumerated, together with four host-associated fecal molecular markers selected to discriminate human, dog, horse, or gull/seagull origins of the contamination. The antimicrobial resistance of bacteria in water and sand was evaluated by quantifying (i) the class 1, 2, and 3 integron integrase genes *intI*, and (ii) *bla*_TEM_, *bla*_CTX–M_, and *bla*_SHV_ genes encoding endemic beta-lactamase enzymes. The number of beach users entering and leaving per hour during the observation period was manually counted. Photographs of the beach and the bathing area were taken every hour and used to count the number of persons in the water and on the sand, using a photo-interpretation method. The number of beach users increased from early morning to a peak by mid-afternoon, totaling more than 1,800, a very large number of users for such a small beach (less than 1 ha). An increase in fecal contamination in the water corresponded to the increase in beach attendance and number of bathers, with maximum numbers observed in the mid-afternoon. The human-specific fecal molecular marker HF183 indicated the contamination was of human origin. In the water, the load of *Intl*2 and 3 genes was lower than *Intl*1 but these genes were detected only during peak attendance and highest fecal contamination. The dynamics of the genes encoding B-lactamases involved in B-lactams resistance notably was linked to beach attendance and human fecal contamination. Fecal indicator bacteria, integron integrase genes *intI*, and genes encoding B-lactamases were detected in the sand. This study shows that bathers and beach users can be significant contributors to contamination of seawater and beach sand with bacteria of fecal origin and with bacteria carrying integron-integrase genes and beta lactamase encoding genes. High influx of users to beaches is a significant factor to be considered in order to reduce contamination and manage public health risk.

## Introduction

In summer, the French Mediterranean coasts record very high attendance due to the existence of numerous beaches which attract tourists and locals for recreational activities related to relaxation (bathing and sunbathing, etc.) and water sports (swimming, snorkeling, kite-surfing, jet-skiing, and paddle boarding, etc.). However, these recreational activities may be reduced and/or prohibited, depending on the sanitary quality of the bathing water.

Those decisions are controlled by relevant authorities when the sanitary quality of the bathing waters does not comply with standards set by the European directive (2006/7/CE-02/2006). These standards rely on two microbiological parameters indicating fecal contamination, thermo-tolerant coliforms (TTC, including *Escherichia coli*) and intestinal enterococci (IE), whose abundance in the water is controlled under the responsibility of the Regional Health Agency (ARS), during the entire summer season but at a variable frequency, depending on the beach. Unlike water, the sanitary quality of the sand is neither controlled nor regulated, and its potential fecal contamination is not considered. When decided, a ban on swimming and nautical activities reduces the number of visitors to beaches and repeated bans may alter the reputation of the seaside resort or the city. It is therefore important to detect and control factors related to poor bathing water quality, to be able to prevent degradation and improve the sanitary condition of the beach.

It has been shown that crowded beaches have high bacterial abundances in both sand and water during hot and sunny summer conditions (Papadakis et al., 1997; Pereira et al., 2013), simply because of the presence of users on the beach. The behavior of users, as well as the sanitary equipment provided by municipalities (showers, toilets, and bins, etc.), can have an impact on the microbiological quality of beach water and sand. In addition, a sanitation network failure resulting in direct discharge of untreated domestic wastewater to coastal waters can be severely detrimental to the quality of bathing water, especially during summer thunderstorms with intense and localized rainfall (Laplace et al., 2009; Figueras et al., 2015). Finally, accidental discharge of sewage from yachts and wild or domestic animal feces are additional factors to consider (Brouwer and De Blois, 2008). Standard microbial indicators do not allow identification of human or animal origin of the fecal contamination. However, in the event of problems with water quality, it is important to determine the origin of the fecal input in order to make management decisions that reduce the sources of contamination. In this perspective, a number of microbial source tracking (MST) methods, were employed, including methodologies developed over the last two decades that target closely related host-specific microorganisms (Roslev and Bukh, 2011).

In this study, a well-recognized and highly attended beach in Marseille, France, was monitored hourly on a summer day to determine water and sand fecal pollution that could be correlated with influx of beach users. Standard microbial indicators were measured to assess the level of fecal pollution (TTC, IE), together with four host-associated fecal molecular markers, chosen in order to discriminate human, dog, horse or gull/seagull origin of the contamination. These trackers targeted the host-specific 16S rRNA gene from *Bacteroidales* that is specific to the fecal microbiota of humans (Bernhard and Field, 2000a, b; Seurinck et al., 2005), dogs (Dick et al., 2005b), and horses (Dick et al., 2005a), and from the bacterium *Catellicoccus marimammalium* specific to seagulls (Lu et al., 2008; Wu et al., 2017). However, because geographical variation in sensitivity and specificity of these tracers has been reported in the literature (Harwood et al., 2014), they were first tested on a regional panel of host feces. Antimicrobial resistance (AMR) is a serious public health concern, therefore the AMR of bacteria in water and sand was determined. This was done by determining: (i) class 1, 2, and 3 integron integrase genes *intI* (Barraud et al., 2010; Gillings et al., 2015) associated with transmission and dissemination of resistance genes in the bacterial community, and (ii) *bla*_TEM_, *bla*_CTX–M_, and *bla*_SHV_ genes, encoding endemic beta-lactamase enzymes that lead to resistance to the beta-lactam antibiotic family (Xi et al., 2009; Salverda et al., 2010; Marti et al., 2013). Finally, the total bacterial load was evaluated by quantifying a conserved region of the 16S rRNA gene (Maeda et al., 2003).

## Materials and Methods

### Study Area and Beach Attendance Assessment

**FIGURE 1:**
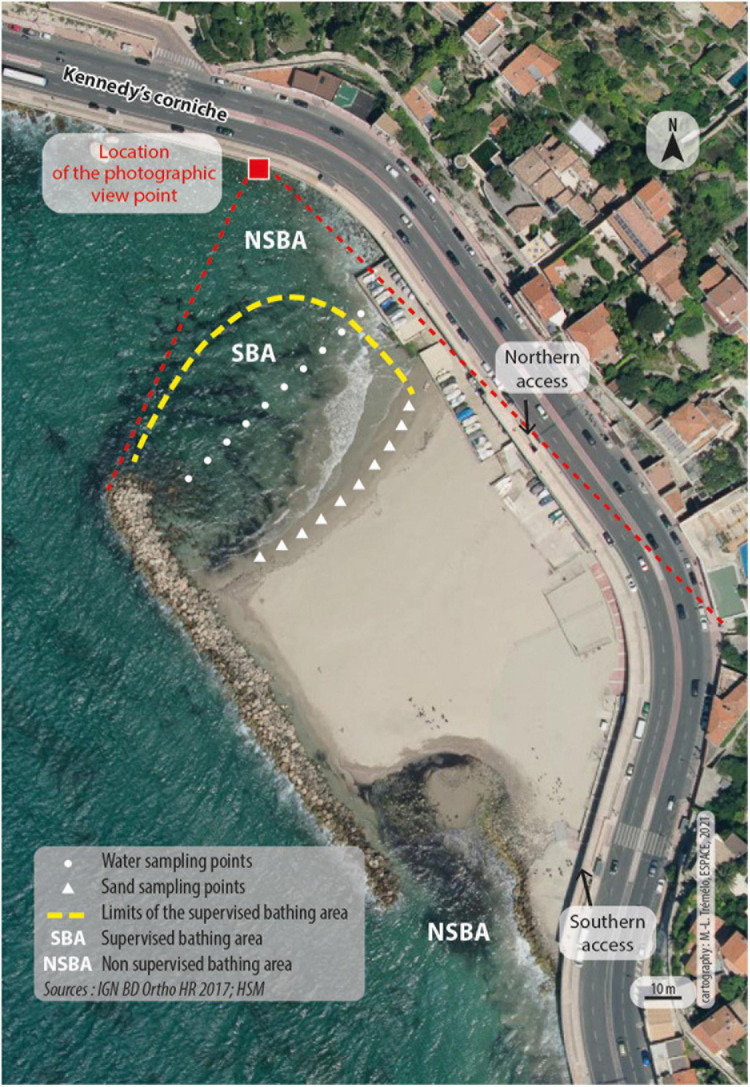
Aerial view of the Prophète Beach in Marseille, Mediterranean coast, South of France. The only two pedestrian accesses by stairs, Northern and Southern, are indicated. On each side of the beach, two bathing areas (BA) are present: a supervised one (SBA) and a non-supervised one (NSBA). In the SBA, ten sampling points separated by 5 m each were determined to collect seawater (white dots) and sand (white triangles). These 10 samples are pooled in one sample for analysis. Map data:© IGN BN Ortho HR 2017; HSM.

Located in Marseille, the second largest town in France (nearly 900,000 inhabitants), the study area was Prophète beach. Backing onto the ledge, this sandy beach is open to the sea on either side of a dike running parallel to the coast and offers two bathing areas, only delineated as a supervised bathing zone (Figure 1). The beach area is approximately 7,000 m^2^. It accommodates a beach volleyball court, a snack bar, toilets, showers, and a small office for bath supervisors. The locale also hosts a snack bar and a restaurant located under the coastal boulevard overlooking the beach.

On July 18, 2018, the number of users at the beach was determined using an observation protocol implemented between 8 a.m. and 8 p.m., i.e., 12 h of continuous observation. Because two stairways are the only entry to the beach by land (Figure 1), this characteristic served to provide a manual count of the number of beach users entering and leaving per hour during the observation period. In addition to this counting, photographic shots of the beach and the bathing area (Figure 1) were carried out every hour and were used to count the number of persons in the water and on the sand, using a photo-interpretation method back at the laboratory (Robert et al., 2008).

### Sampling and Processing of Water and Sand Samples

#### Sampling and Numeration of Fecal Indicator Bacteria

Samples of approximately 200 mL of water collected at each of ten sampling points spaced evenly across the supervised bathing area were combined in a sterile bottle (Figure 1). Sand was collected at ten points located at the water edge in the area of the surf. These points were spaced evenly across the beach. Sand samples were obtained using a sterile container to a final weight of approximately 250 g. At the time of sampling, the bathing water temperature was measured. All samples were stored in refrigerated coolers for immediate processing. Analyses were done in a field laboratory installed in a prefab module provided by the City of Marseille and located at the Roucas Blanc Marina near Prophet Beach (approximately 1 km).

Fecal indicator bacteria (FIB) in the water were enumerated using reference methods ISO 9308-1 and ISO 7899-2 referenced in the European bathing water directive 2006/7/EC to enumerate thermo-tolerant coliforms (TTC) and IE, respectively (European Parliament, 2006). Water volumes (1, 10, and 100 mL) were filtered in duplicate on 0.45 μm pore size cellulose nitrate filters (Sartorius). TTC were quantified on triphenyl tetrazolium chloride and tergitol 7 lactose agar (Biokar Diagnostics) incubated for 24 h at 44.5°C, and IE were enumerated on Slanetz medium (Biokar Diagnostics) incubated for 48 h at 37°C. The results are expressed as colony forming units per 100 mL (CFU/100 mL).

For sand, the sample was thoroughly mixed and 100 g was suspended in a bottle with 400 mL of sterile water and shaken to release the bacteria. After a settling time (1 min) of the larger particles, 4 × 100 mL of the wash water was filtered on 0.45 μm pore size cellulose nitrate filters (Sartorius). Two filters were used to count TTC and two filters for IE. This step was repeated three times in order to extract the bacteria. After incubation, eight membranes were counted for TTC or for EI to obtain the CFU for 100 g of sand.

The extraction protocol for sand is time consuming and tedious. Also, measurement of FIB in sand was done three times at 8 a.m., midday, and 5 p.m., while the measurements for the water samples were hourly, from 8 a.m. to 8 p.m.

#### Total DNA Extraction

Seawater samples: 500 mL of each composite water sample was filtered in triplicate through 0.2 μm pore size cellulose acetate filters (Sartorius) and filters were stored immediately at −20°C. Sand samples: 3 × 50 g of each composite sample was suspended in 200 mL of sterile water in three different bottles and were shaken to release the bacteria. The washing water was filtered using a 0.2 μm pore size cellulose acetate filter (Sartorius). Three more washes of the same three biological replicates of sand were made under the same conditions, and the wash water was filtered on the same cellulose acetate filter. In total, each biological replicate of 50 g of sand was washed using 800 mL of sterile water. Filters were immediately stored at −20°C until processing.

At the laboratory, total genomic DNA was extracted from filters using DNeasy PowerWater kit (Qiagen) according to the manufacturer’s instructions and eluted in a final volume of 100 μL. Concentrations were measured on a Qubit fluorometer (Invitrogen) using the Qubit dsDNA HS assay kit (Invitrogen) following the manufacturer’s instructions, and the DNA was stored at −20°C until qPCR analysis.

### Fecal Sample Collection to Test for Host-Associated Molecular Markers

Four host-associated molecular markers were selected to determine the origin of fecal contamination: the human-specific HF183 *Bacteroides*-related, the dog-specific DF475 *Bacteroides*-related, the horse-specific HoF597 *Prevotella*-related and the gull/seagull-specific bacterium *Catellicoccus marimammalium* (Table 1). These markers were first tested using local fecal samples collected in 2018 in the Occitanie region, South of France, to determine sensitivity and specificity. Amplification tests were done by Real-time quantitative PCR (qPCR) on DNA extracted from fecal samples from 51 birds (11 species), including 19 gulls and seagulls, collected by the “Ligue de Protection des Oiseaux” (Hérault), from six dogs, collected by owners (Montpellier, Hérault) and from 15 horses, collected at a riding stable (Saint-Affrique, Aveyron). For birds, feces were sampled using a sterile swab, suspended immediately in 1 mL of sterile water and stored at 4°C. For dogs and horses, feces were collected directly in sterile tubes and stored at −20°C. Total DNA was purified from 1 mL of a feces suspension (birds) or from 50 mg of wet feces (dogs and horses), using the MasterPure^TM^ Gram positive DNA purification kit (Epicentre) according to the supplier instructions (starting with feces pellet instead of bacteria). A total of 55 DNA extracts from human fecal samples (anonymous patients) were obtained from a university hospital center. DNA extractions were obtained using a Seegene Nimbus Automated Liquid Handling Workstation. Quantities and qualities of DNAs were measured by spectrophotometry using a NanoDrop One (Thermo Scientific). All DNA samples were stored at −20°C until used.

**TABLE 1:** Primers and conditions used for end-point and real-time PCR quantifications.

**Target**	**Primers (5′–>3′)**	**Final concentration (μM)**	**Amplicon size (bp)**	**Annealing temperature (°C)**	**References**
**Total bacteria**					
*16S rRNA gene*	16SrRNA-F GTGSTGCAYGGYTGTCGTCA	0,4	147*	60	Maeda et al., 2003
	16SrRNA-R ACGTCRTCCMCACCTTCCTC	0,4			
**Host-associated fecal molecular markers**					
Human-specific *Bacteroides*-related HF183	HF183F ATCATGAGTTCACATGTCCG	0,4	83	60	Seurinck et al., 2005
	HF183R TACCCCGCCTACTATCTAATG	0,4			
Dog-specific *Bacteroides*-related DF475	DF475F CGCTTGTATGTACCGGTACG	0,4	251	56	Dick et al., 2005b
	Bac708R CAATCGGAGTTCTTCGTG	0,4			
Horse-specific *Prevotella*-related HoF597	HoF597F CCAGCCGTAAAATAGTCGG	0,5	127	56	Dick et al., 2005a
	Bac708R CAATCGGAGTTCTTCGTG	0,5			
Gull/Seagull-specific Sg2	Sg2-F TGCTAATACCGCATAATACAGAG	0,4	306	60	Wu et al., 2017
	Sg2-R CTATCGCTCCTGTTCTTCTCTAA	0,4			
**Integron integrase genes**					
*Class 1-Intl1*	intI1-LC1 GCCTTGATGTTACCCGAGAG	0,4	196	60	Barraud et al., 2010
	intI1-LC5 GATCGGTCGAATGCGTGT	0,4			
*Class 2–Intl2*	intI2-LC2 TGCTTTTCCCACCCTTACC	0,4	195	62	Barraud et al., 2010
	intI2-LC3 GACGGCTACCCTCTGTTATCTC	0,4			
*Class 3–Intl3*	intI3-LC1 GCCACCACTTGTTTGAGGA	0,4	138	62	Barraud et al., 2010
	intI3-LC2 GGATGTCTGTGCCTGCTTG	0,4			
**Beta-lactam resistance genes**					
*bla*_TEM_	bla-TEM-F GCKGCCAACTTACTTCTGACAACG	0,4	247	60	Xi et al., 2009
	bla-TEM-R CTTTATCCGCCTCCATCCAGTCTA	0,4			
*bla*_SHV_	bla-SHV-F CGCTTTCCCATGATGAGCACCTT	0,4	110	60	Xi et al., 2009
	bla-SHV-R TCCTGCTGGCGATAGTGGATCTT	0,4			
*bla*_CTX–M_	bla-CTX-M-F CTATGGCACCACCAACGATA	0,4	103	62	Marti et al., 2013
	bla-CTX-M-R ACGGCTTTCTGCCTTAGGTT	0,4			

*Length of the amplicon obtained with positive control bacterial strain.

### Gene Quantifications by Real-Time Quantitative PCR

#### Quantitative PCR Reactions

The absolute number of genes was determined by real-time PCR (qPCR) in 96-well plates using a LightCycler^®^ 480 (Roche). Each qPCR reaction employed Syber Green, was run in duplicate, and contained 1X Luna^®^ Universal qPCR Master Mix (New England BioLabs), 0.4–0.5 μM of each specific primer (Table 1), 1 μL of sample DNA (extracted from a fecal sample, seawater, or sand), and sterile water to a final volume of 10 μL. Sterile water was used instead of DNA as the negative control. After heating for 10 min at 95°C for activation of the hot-start DNA polymerase and denaturation of DNA, reactions were carried out for 45 cycles of 95°C for 10 s, 56–62°C for 10 s, and 72°C for 10 or 15 s (depending on the amplicon size, Table 1). Finally, qPCR products were gradually heated from 65 to 95°C to obtain a dissociation curve in order to determine the temperature of melting and the specificity of the amplicons. Bacterial type strains *Bacteroides dorei* (DSM 17855, DSMZ) and *Catellicoccus marimammalium* (DSM 18331, DSMZ) were used as positive control for the human-specific and the gull/seagull-specific markers, respectively. Environmental and clinical multi-drug resistant *Klebsiella pneumoniae* and *E. coli* strains (isolated from previous studies in Montpellier city, France) were used as positive control for total 16S rRNA, *intl* and *bla* genes. Total DNA was extracted from fresh cultures using the MasterPure^TM^ Complete or Gram-positive purification kit (Epicenter) according to the manufacturer instructions. For dog and horse associated markers, DNA from two positive fecal samples was used as positive control.

#### Standard Curves and Determination of the Limits of Quantification

All specific amplicons were sequenced to ensure their identity (Genewiz, Germany) and cloned using TOPO^®^ TA cloning^®^ kit (Invitrogen) with pCR^TM^4-TOPO^®^ cloning vector, according to the manufacturer’s instructions. After cloning, plasmids were purified using NucleoSpin Plasmid kit (Marcherey-Nagel) and linearized by enzymatic digestion using *Pst*1 (New England Biolabs). Finally linearized plasmids were purified using Monarch PCR and DNA Cleanup Kit (New England BioLabs), and concentrations measured on spectrophotometer NanoDrop One (Thermo Scientific) in order to calculate the number of plasmid copies per microliter. Then, 10-fold serial dilutions of each plasmid were made in 10 μg/mL sonicated salmon sperm DNA (Sigma) and these dilutions were used in qPCR to determine the standard curves, to establish the amplification efficiencies and to calculate the absolute quantifications of the genes in environmental samples. Based on standard curve results, the limits of quantification (LOQ) of the assays were determined for each gene as the lowest concentration of marker within the linear range of quantification during amplification.

### Calculations and Statistics

The sensitivity (percentage of true positives among fecal samples of the targeted hosts) and specificity (percentage of true negatives among fecal samples of non-targeted hosts) were calculated for each host-associated molecular marker according to qPCR results.

For gene quantifications, results were transformed in number of gene copies for 1 mL of water sample (GC/mL) (or for 1 g of wet sand) and the final result was calculated as the arithmetical mean of the three biological replicates. The relative quantities of targeted genes were calculated as the ratio between the number of copies of each gene to the total number of 16S rRNA gene copies, in order to control the reproducibility and quality of DNA extractions and normalize their abundance in the collected samples.

The normality of the data distributions was tested by using a Shapiro-Wilk test, and equality of variances tested by using a Fisher test. In order to determine the correlations between variables, the Spearman’s rank correlation coefficients (rs) were calculated and their nullity tested. All statistics were done using the GraphPad Prism software V 5.03. Test results were considered significant when the associated *p*-value was at least ≤0.05.

## Results

### Beach User Influx on July 18, 2018

Weather conditions on July 18, 2018, were typical still air and sunny summer day. The average daily temperature from the nearby Marseille Corniche weather station was 26°C, bathing water temperature in the morning was 22°C and warmed to close to 25°C in the afternoon.

**FIGURE 2:**
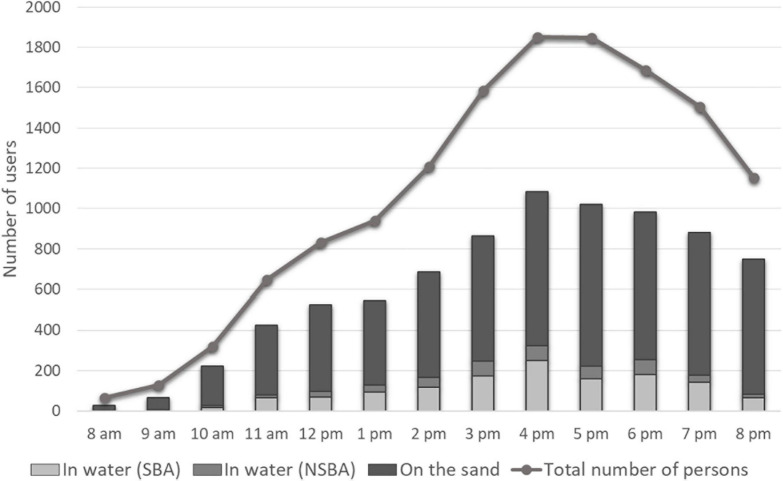
Influx of users on July 18th 2020 on the Prophète beach. Number of users in the water in the supervised and non-supervised bathing areas (SBA and NSBA) and number of users on the sand were determined by photo-interpretation. Total number of persons was determined by the counts of entries and exits at the two only accesses (stairs).

The total number of persons counted at the two entrances of the beach between 8 a.m. and 8 p.m. was 5,514 (Robert and Trémélo, 2019). This attendance is the highest ever at the Prophète beach, where such counts have been made every summer in July since 2016. As shown in Figure 2, the number of persons counted from the hourly photographs was lower than the count at the two entrances, which is not surprising. In photos, users can be hidden by beach umbrellas, tents, or other users, in particular when the beach is overcrowded. In water, counts made from the photographs are more reliable as the bathing zone is at the foreground, improving identification of individuals.

From counts at the two entrances, the number continued to increase from 8 a.m. to 4 p.m., reaching a little approximately 1,800 persons between 4 p.m. and 5 p.m. In the late afternoon and early evening, attendance decreased but remained high, with nearly 1,200 persons still at the beach at 8 p.m. From the photographs, users in the water in both the supervised and non-supervised bathing areas (SBA/NSBA) were present at a rather high level also. Between 2 p.m. and 7 p.m., more than 150 bathers could be counted every hour, reaching a maximum of 322 persons at 4 p.m., with more than two thirds in the supervised bathing area. During the attendance peak, nearly one beach user of six was in the water.

### Fecal Indicator Bacteria Levels in Seawater and Sand

Quantities of TTC and IE numerated in seawater and sand are shown in Table 2. Concerning TTC, levels varied throughout the day in seawater, with low quantities (60–160 CFU/100 mL) from 8 a.m. to 3 p.m., and high levels estimated at over 500 CFU/100 mL (uncountable) from 4 p.m. to 8 p.m. These TTC loads exceeded the limits determined by the European directive 2006/7/CE-02/2006 (500 *E. coli*/100 mL) for sufficient water quality for bathing. In the sand, quantities were 44 TTC/100 g at 8 a.m., 108 at midday, and 360 at to 5 p.m.

**TABLE 2:** Fecal indicator bacteria (FIB) levels in seawater and sand.

	**Thermo-tolerant coliforms**		**Intestinal enterococci**	
**Hours of sampling**	**Seawater (CFU/100 mL)**	**Wet sand (CFU/100 g)**	**Seawater (CFU/100 mL)**	**Wet sand (CFU/100 g)**
8 a.m.	160	44	67	326
9 a.m.	135	–	72	–
10 a.m.	N.A.	–	63	–
11 a.m.	N.A.	–	60	–
Midday	N.A.	108	54	270
1 p.m.	70	–	67	–
2 p.m.	65	–	93	–
3 p.m.	60	–	30	–
4 p.m.	>*500*	–	47	–
5 p.m.	>*500*	360	37	214
6 p.m.	>*500*	–	73	–
7 p.m.	>*500*	–	64	–
8 p.m.	>*500*	–	64	–

*For seawater, quantities above the thresholds for sufficient quality for bathing are indicated in italics.*

*N.A., not accountable; –, not measured.*

Concerning IE, quantities remained low in seawater during the day, with variations from 30 to 93 IE/100 mL. These results did not exceed 185 IE/100 mL which is the limit for sufficient water quality for bathing. In the sand, quantities were 326 IE/100 g at 8 a.m., 270 at midday, and 214 at to 5 p.m.

### Origin of the Fecal Contamination

#### Sensitivity and Specificity of Host-Associated Fecal Molecular Markers

The detection results, obtained by qPCR, of host-associated fecal molecular markers in fecal samples are provided in Table 3. Of the 55 human fecal samples, 31 were positive for presence of the human-specific HF183 marker, which established its sensitivity at 56.36%. This marker was never detected in non-human fecal samples. Therefore, HF183 marker displayed moderate sensitivity of 56.36% and a high specificity of 100%. Dog-specific DF475 marker showed 83.33% sensitivity and 98.34% specificity due to its detection in two gull feces samples. Horse and gull/seagull-specific markers were both highly sensitive (100%) and highly specific (99.1 and 96.29%, respectively). Indeed, the horse-specific marker was found in one gull fecal sample, and the gull/seagull marker was detected in four fecal samples from other bird species. These tests were done on a small number of pure fecal samples. Nevertheless, due to their high sensitivity and specificity, the use of these four markers was validated to quantify the origin of the fecal pollution in environmental samples in this study.

**TABLE 3:** Sensitivity and specificity of host-associated fecal molecular markers.

	**Host-associated fecal molecular markers**			
**Samples**	**Human-specific HF183**	**Dog-specific DF475**	**Horse-specific HoF597**	**Gull/Seagull-specific Sg2**
Human (*n* = 55)	31/55 (56,36%)	0/55 (0%)	0/55 (0%)	0/55 (0%)
Dog (*n* = 6)	0/6 (0%)	5/6 (83,33%)	0/6 (0%)	0/6 (0%)
Horse (*n* = 15)	0/15 (0%)	0/15 (0%)	15/15 (100%)	0/15 (0%)
Gull/Seagull (*n* = 19)	0/19 (0%)	2/19 (10,53%)	1/19 (5,26%)	19/19 (100%)
Other birds (*n* = 32)	0/32 (0%)	0/32 (0%)	0/32 (0%)	4/32 (12,5%)
Sensitivity (r)	56,36%	83,33%	100,00%	100,00%
Specificity (s)	100,00%	98,34%	99,10%	96,29%

*For each marker, the number of positive samples obtained by qPCR among each panel of samples are given, and corresponding percentages are indicated into brackets.*

#### Quantification of Total 16S rRNA Gene and Host-Associated Fecal Molecular Markers in Seawater and Sand

The qPCR amplification efficiencies and LOQ obtained for the four host-associated fecal molecular markers and the total 16S rRNA gene are reported in Supplementary Table 1. Amplification efficiencies were excellent, ranging from 92 to 96.9%. The lowest LOQs were for the dog, horse and gull/seagull-specific markers, with a limit of 10 gene copy number (GC) per reaction, corresponding to 2 GC/mL of seawater and 20 GC/g of sand. The LOQ for the human-specific marker was a little higher, with 50 GC per reaction, corresponding to 10 GC/mL of seawater and 100 GC/g of sand. Finally, the highest LOQ was for total 16S rRNA gene, with a limit of 60 GC reaction, corresponding to 12 GC/mL and 120 GC/g of sand.

**FIGURE 3:**
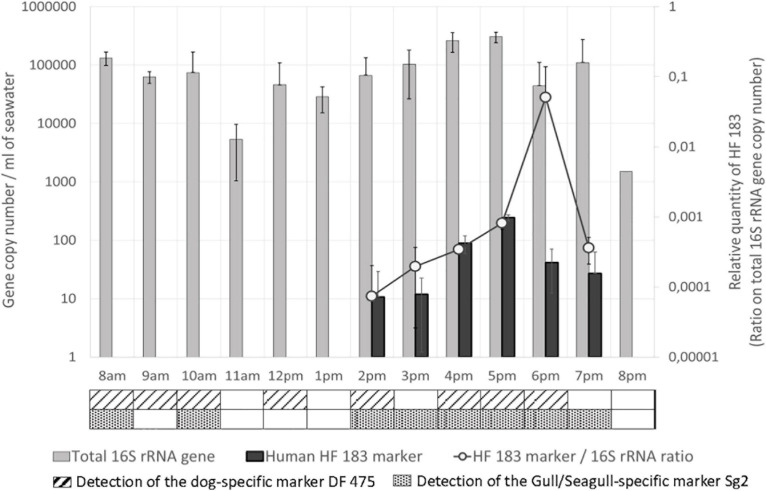
Total 16S rRNA gene and host associated molecular markers levels in seawater. Quantities are given in gene copy number for 1 mL of seawater for the total 16S rRNA gene and the human-specific associated marker HF 183. Relative quantities of HF 183 are indicated on the secondary Y-axis. The detection (<Limit of quantification, LOQ) of the dog and the gull/seagull-specific associated markers are indicated in the table below the graph.

In seawater, the total number of 16S rRNA gene, representative of the total bacterial load, fluctuated during the day (Figure 3): levels first decreased during the morning from 8 a.m. (1.32 × 10^5^ GC/mL) to 11 am (5.37 × 10^3^ GC/mL), and then increased from 12 p.m. (4.59 × 10^4^ GC/mL) to reach a maximum value of 3.05 × 10^5^ GC/mL at 5 p.m. Levels then decreased until 8 p.m. (1.5 × 10^3^ GC/mL). In the sand, the highest quantities were measured at 8 a.m., with 6.63 × 10^5^ GC/g. The total load decreased at midday, with 3.87 × 10^4^ GC/g, and increased at 5 p.m. to 2.69 × 10^5^ GC/g (data not shown).

The human-specific fecal molecular marker HF183 (Figure 3) was first detected in seawater at 2 p.m. (10.6 GC/mL), and slowly increased until 5 p.m. (243 GC/mL). Levels then decreased from 5 p.m. to 7 p.m. (27 GC/mL) and the marker was not detected anymore at 8 p.m. Relative quantities of this marker (ratio on the total number of 16S rRNA gene copies), representative of its abundance in total bacterial community, increased in the same way during the afternoon from 2 p.m. to a maximum at 6 p.m., and finally decreased until 7 p.m. In the sand, the human-specific marker was only detected at 5 p.m. (<LOQ). The quantities of the human-specific marker in seawater were strongly positively correlated with the total number of persons at the beach (rs = 0.908, *p* < 0.001), in the water (rs = 0.872, *p* < 0.001) and on the sand (rs = 0.89, *p* < 0.001), with maximum values occurring between 4 p.m. and 6 p.m., when the number of persons at the beach was also maximum. This marker was also correlated with the total bacterial load (rs = 0.603, *p* < 0.05).

The presence of the dog and gull/seagull-specific markers were detected (<LOQ) in seawater approximately between 8 a.m. and 10 a.m., and between 2 p.m. and 6–7 p.m. (Figure 3). The dog-specific marker was also detected at midday. These two markers were absent in the sand. Finally, the horse-specific marker was not detected either in seawater or in the sand.

### Antimicrobial Resistance of Bacteria in Seawater and Sand

The qPCR amplification efficiencies and LOQ for the integron-integrase genes (*Intl*1, 2, and 3) and beta lactamase encoding genes (*bla*_TEM_, *bla*_SHV_, and *bla*_CTX–M_) are reported in Supplementary Table 1. The amplification efficiencies were also excellent, ranging from 96.1 to 102.4%. Efficiencies over 100% were surely due to minor pipetting errors but stayed in a range of good quality efficiencies. The LOQ for these six genes were 5 GC per reaction, which corresponded to 1 GC/mL of seawater and 10 GC/g of sand.

#### Quantification of Integron Integrase Genes (*Intl*)

**FIGURE 4:**
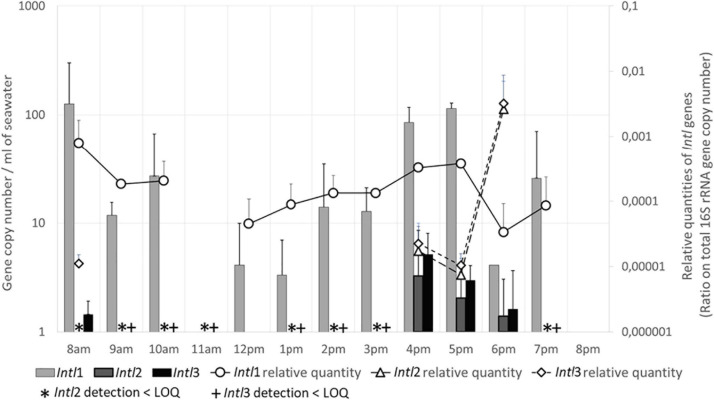
Integron integrase genes (*Intl*) levels in seawater. Quantities are given in gene copy number for 1 mL of seawater. Relative quantities of *Intl* genes are indicated on the secondary Y-axis. LOQ, limit of quantification.

In seawater, *Intl*1 gene was present almost all the day, except at 11 a.m. and at 8 p.m. (Figure 4). Measured quantities varied from 125 GC/mL at 8 a.m. to 27 GC/mL at 10 a.m., and increased gradually in the afternoon from 5 GC/mL at midday to a maximum of 114 GC/mL at 5 p.m. Densities then decreased at 6 p.m. and 7 p.m. (12 and 25 GC/mL), until no longer detected at 8 p.m. *Intl*1 gene density was higher in sand than in seawater. A maximum of 3,823 GC/g was detected at 8 a.m. Densities then decreased significantly at midday, with only 9 GC/g, and then increased again to 853 GC/g at 5 p.m. The relative amounts of *Intl*1 gene varied accordingly with the GC/mL in seawater (Figure 4) and with the GC/g in the sand (data not shown). Moreover, the quantities of *Intl*1 gene and of total 16S rRNA gene were strongly correlated (rs = 0.927, *p* ≤ 0.001), which suggested that the variations in *Intl*1 gene quantities were proportional to the total bacterial concentration.

The density of *Intl*2 and *Intl*3 genes was significantly lower than *Intl*1 gene. The dynamics and load of both *Intl*2 and *Intl*3 genes in seawater were similar (Figure 4) and were significantly correlated (rs = 0.903, *p* ≤ 0.001): both genes were detected (1 GC/mL or below the LOQ) from 8 to 11 a.m. and from 1 to 3 p.m. Both became quantifiable from 4 to 6 p.m., with quantities varying from 3 (*Intl*2) and 5 GC/ml (*Intl*3) to 1 GC/mL. These genes were still present at 7 p.m. but not at 8 p.m. In the sand, *Intl*2 and *Intl*3 genes were only slightly numerous (<LOQ) at 8 a.m. and in the afternoon, respectively. When regarding the relative abundance of both *Intl* 2 and 3 genes in the total bacterial community in seawater (Figure 4) a huge increase was observed at 6 p.m. but this could be artificial due to the very small quantities of *Intl* 2 and 3 genes measured. It is worth mentioning that the quantities of these two genes were significantly correlated with quantities of the human-specific marker HF183 (rs = 0.796 and 0.664, *p* ≤ 0.001 and 0.05), and also for *Intl*2 gene to the number of persons in the water and on the sand (rs = 0.684 and 0.739, *p* ≤ 0.01).

#### Quantification of Beta Lactamase Encoding Genes (*bla*)

**FIGURE 5:**
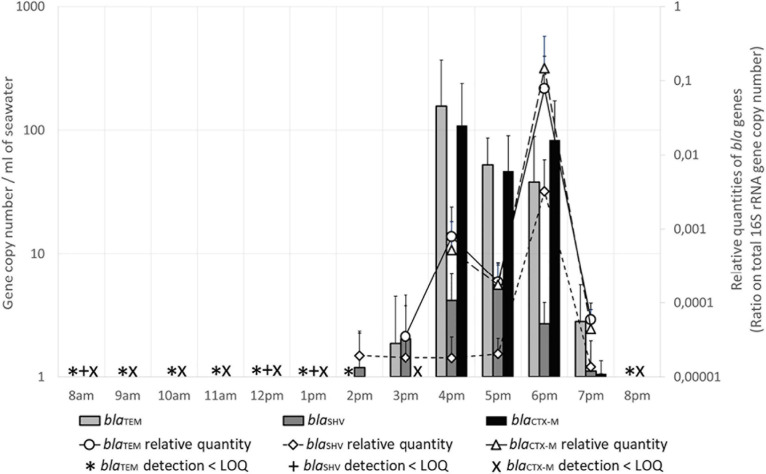
Beta lactamase encoding genes (*bla*) levels in seawater. Quantities are given in gene copy number for 1 mL of seawater. Relative quantities of *bla* genes are indicated on the secondary Y-axis. LOQ, limit of quantification.

The variations of the quantities of *bla*_TEM_ and *bla*_CTX–M_ genes in seawater were almost the same during the day (Figure 5): these genes were present (<LOQ) from 8 a.m. to 2 p.m. (*bla*_TEM_) or to 1 p.m. (*bla*_CTX–M_). At 3 p.m., *bla*_CTX–M_ was still present and *bla*_TEM_ began to be quantifiable, with almost 2 GC/mL. At 4 p.m., the measured quantities of both genes were maximum, with 157 and 110 GC/mL for *bla*_TEM_ and *bla*_CTX–__M_ respectively, then the numbers slightly decreased to 39 and 84 GC/mL at 6 p.m., respectively. At 7 p.m., the numbers were much lower (1–3 GC/mL), and both genes were only detectable at 8 p.m. The proportion of these genes in the total flora (relative quantities, Figure 4) was increasing at 4 p.m., and was maximum at 6 p.m. In the sand, *bla*_TEM_ gene quantity was 14 GC/g at 8 a.m., and both genes were detected (<LOQ) all day.

The numbers of *bla*_SHV_ gene were much lower in seawater (Figure 4): it was detected only at 8 a.m., midday and 1 p.m., and was quantifiable between 3 p.m. to 7 p.m. (1 GC/mL) with a maximum value at 5 p.m. with 6 GC/mL. The relative number was maximum at 6 p.m. In the sand, this gene was detected only at 8 a.m.

The number of these three *bla* genes in seawater was positively correlated (rs = 0.781–0.926, *p* ≤ 0.01–0.001). Maximum numbers were recorded between 4 and 6 p.m., when the number of users at the beach, the levels of the human-specific marker HF183, and the number of *Intl*2 and *Intl*3 genes were also at their maximum. Moreover, all these parameters were significantly correlated (rs = 0.62–0.947, *p* ≤ 0.05 to ≤0.001).

## Discussion

The study was carried out during a very busy summer day at the Prophète Beach, an urban beach located in Marseille, a very large city. The number of beach users increased from early morning to a peak by mid-afternoon, as generally reported in other studies of beach attendance in summer time (Robert et al., 2008; Balouin et al., 2014). The peak of users in the middle of the afternoon was particularly significant (more than 1,800 people) for such a small beach (less than 1 ha).

The increase in fecal contamination (TTC) in the water followed the increase in beach attendance and bathers with maximum numbers of users observed mid-afternoon. However, the concentration of IE remains stable and does not increase with increasing TTC. The relationship between IE and TTC concentrations is not a rule. It has been shown that the concentrations of enterococci in the coastal estuarine/marine beach study area were largely controlled by particle-associated enterococci and mammal fecal source input (Rothenheber and Jones, 2018). The human-specific marker indicates that contamination was of human origin. The presence of bird- and dog-specifics markers was very low and did not contribute significantly to the fecal contamination observed in the water.

We demonstrated that monitoring fecal indicators and specific markers throughout the day and in presence of people using the beach provides valuable information for evaluating public health risk. Indeed, weekly to bi-weekly monitoring of water quality by health authorities is usually accomplished in the early morning when the beach is sparsely attended. The day this study was done, the Prophète beach was open, which means that surveillance showed the microbiological quality of the water was within the limits of the European directive. However, the mid-afternoon peak exceeded those limits and exposed bathers to potential health risk. The observed concentrations of *Bacteroides*-related HF183 marker represent a health risk for bathers as shown by Boehm et al. (2015). The dynamics of fecal indicators throughout the day could help define policies and/or remediation practices for more safe beach use.

Remediation efforts can be hindered because classical water quality surveillance does not take into account whether contamination originated from humans or animals. We determined the most likely origin of the fecal contamination by using markers previously described as efficient for origin tracing (Hughes et al., 2017). In particular, *Bacteroides*-related HF183 marker is considered the most sensitive measure of human fecal pollution (Hughes et al., 2017).

The analysis used host-associated markers of fecal samples and showed 100% specificity and 56.36% sensitivity for the human-specific *Bacteroides*-related HF183 marker. This sensitivity for the HF183 marker is consistent with other studies in France, with 54 and 62.5% sensitivity observed in Brittany (Mieszkin et al., 2009; Mauffret et al., 2012), and in other countries, e.g., in California, United States (Kildare et al., 2007), and in Kenya, Africa (Jenkins et al., 2009) with 61.1 and 58.3% sensitivity, respectively. For dog-specific *Bacteroides*-related DF475, horse-specific *Prevotella*-related HoF597, and Gull/Seagull-specific Sg2 markers, the results showed very high specificity and sensitivity. So, these four markers proved to be efficient and of great interest for fecal contamination tracking (Roslev and Bukh, 2011; Tran et al., 2015).

Various studies have shown that bathers contribute to contamination of water, namely with *Staphylococcus aureus*, enteric viruses, protozoan parasites, IE, and fecal coliforms (Breittmayer and Gautier, 1978; Gerba, 2000; Elmir et al., 2007). In particular, it has been shown that bathers may develop enteric infections from contamination with stools of other bathers (Keene et al., 1994). It has been shown that the behavior of toddlers can be a main cause of contamination. Indeed, during this study, we observed parents encouraging children under 5 to urinate and defecate in the water, and others collected baby diapers after wringing them out in seawater. Gerba (2000) showed in a review of the literature that bathers of all ages shed enteric microorganisms through normal recreational water contact or accidental fecal release. In our study of the Prophète beach, the same issues were highlighted. Overcrowding of such a small beach can lead to fecal contamination of bathing water, very likely caused by bathers.

Antibiotic resistance is a rising threat for human health and it is now recognized that a one-health based approach should be used to understand, prevent and treat AMR. In this context, spread in the marine environment is poorly understood. Therefore, besides the risk of contamination of recreational water by microorganisms able to cause enteric infections, the presence of human-originating bacteria raises the question of AMR and the potential role of recreational waters in diffusion of AMR. In this study, we tested a recognized marker of AMR in the environment, i.e., class 1 integron-integrase gene (*Intl*1) (Gillings et al., 2015). The *Intl*1 gene is widely used as a proxy for anthropogenic pollution (Gillings et al., 2015). Other genes, such as the sulphonamide resistance gene (*sul*2), are classical markers for AMR. It is noteworthy, that the intI1 gene is detected only in a small fraction of drug-resistant bacteria of health concern (Rodríguez et al., 2021) and the *sul*2 gene conferred resistance to a class of antimicrobial agents practically unused today in human medicine. For these reasons, we detected in this study the *Intl*2 and 3 genes, as well as three genes conferring resistance to B-lactams, the most used class of antibiotics in human and animal medicine. The *Intl*1, 2, and 3 genes are described as frequently occuring in the terrestrial environment and less frequently in the sea (Abella et al., 2015). In the water at Prophète Beach, the *Intl*1 load was not totally correlated with human presence or human fecal contamination. The load of *Intl*2 and 3 genes was lower than of *Intl*1 but these genes were detected only during peak attendance at the beach and with fecal contamination. This suggests the *Intl*2 and 3 genes may be more specific than *Intl*1 to detect human contamination in marine beach areas. *Intl*1 has been reported to be present in plankton-associated bacterial communities in the ocean (Di Cesare et al., 2018) and involved in bacterial adaptation not only to antimicrobial agents but also to hydrocarbon and metals (Gillings et al., 2015). Urban pollution of Prophète Beach may explain the high load of *Intl*1 and its dynamics, which is not fully correlated with human attendance.

The dynamics of genes encoding B-lactamases involved in B-lactam resistance was observed to be correlated with attendance and human fecal contamination. This important result suggests B-lactamass encoding genes may be valuable markers for AMR in the environment and also for human fecal contamination. B-lactamases encoding genes may also provide data for environment-based epidemiology because they are involved in resistance to the most used class of antibiotics. For instance, the load of *bla*_CTX–M_ observed mid-afternoon at the Prophète Beach is of particular concern because *bla*_CTX–M_ encodes an extended spectrum B-lactamase that confers multi-drug resistance to the enterobacteria. The spread of *bla*_CTX–M_, first in hospital and then in the community, is supposedly because extended spectrum B-lactamases are increasingly detected in human or animal infections caused by enterobacteria. A high load of *bla*_CTX–M_ in a water environment integrating contamination from numerous individuals demonstrated spread of *bla*_CTX–M_ within the entire population, including healthy individuals.

Fecal indicator bacteria, integron integrase genes *intI*, and genes encoding B-lactamases were present in the sand. There is some work on the presence and quantification of FIB in sand (Wheeler Alm et al., 2003; Yamahara et al., 2012; Sabino et al., 2014; Whitman et al., 2014). Although contamination in sand is of increasing interest, bathing regulations are based only on the quality of bathing water. In addition, there are no standard methods for monitoring and comparing results obtained around the world. The results of this study show that sand can contribute to contamination by beach users. However, more work needs to be done to understand this relationship between water and sand contamination.

This study shows that bathers and beach users are significant contributors to the contamination of seawater and beach sand with bacteria of fecal origin and also with bacteria carrying integron-integrase genes and beta lactamase encoding genes. This finding contributes to the understanding of an epidemiological risk that is not currently taken into account in sanitary approaches to the quality of bathing water and beaches. At present, the current rules and laws for managing the sanitary quality of bathing water are based on studies that consider possible sources of contamination as only from the beach watershed, particularly during heavy rains, as is the case for the urban beach of Marseille (Laplace et al., 2009). Our study concludes that high influx of users on beaches must be taken into account because beach users and bathers also may contribute to sea water and beach sand microbial contamination.

## Data Availability Statement

The raw data supporting the conclusions of this article will be made available by the authors, without undue reservation.

## Author Contributions

M-GT, CS, SR, and PM led on conceptualization of the study and participated in the sampling of seawater and sand. M-LT and SR carried out the counts of the users of the beach. MT, PL-F, IZ, and PM carried out the microbiological and molecular biology analyzes. All authors contributed to the article and approved the submitted version.

## Conflict of Interest

The authors declare that the research was conducted in the absence of any commercial or financial relationships that could be construed as a potential conflict of interest.

## Publisher’s Note

All claims expressed in this article are solely those of the authors and do not necessarily represent those of their affiliated organizations, or those of the publisher, the editors and the reviewers. Any product that may be evaluated in this article, or claim that may be made by its manufacturer, is not guaranteed or endorsed by the publisher.
