# Supplementary material for: Monitoring Urban Beach Quality on a Summer Day: Determination of the Origin of Fecal Indicator Bacteria and Antimicrobial Resistance at Prophète Beach, Marseille (France)
Source: Front Microbiol. 2021 Aug 25;12:710346. doi: 10.3389/fmicb.2021.710346 (PMC8424182; doi:10.3389/fmicb.2021.710346)
Supplement: Supplementary file 1 [file Table_1.DOCX]

**Supplementary Table 1.** Amplification efficiencies and limits of quantification (LOQ) for the targeted genes.

| Gene | Amplification efficiencies (%) | Limit of quantification (LOQ)  (gene copies number) | | |
| --- | --- | --- | --- | --- |
|  |  | Per reaction (1 µl of DNA) | Per ml of seawater | Per g of sand |
| *16S rRNA* | 96,9 | 60 | 12 | 120 |
| Human-HF 183 | 96,8 | 50 | 10 | 100 |
| Dog-DF 475 | 95,8 | 10 | 2 | 20 |
| Horse-HoF 597 | 94,1 | 10 | 2 | 20 |
| Gull/Seagull-Sg2 | 92 | 10 | 2 | 20 |
| *Intl1* | 96,1 | 5 | 1 | 10 |
| *Intl2* | 96,7 | 5 | 1 | 10 |
| *Intl3* | 102,4 | 5 | 1 | 10 |
| *bla*_TEM_ | 99,5 | 5 | 1 | 10 |
| *bla*_SHV_ | 100,8 | 5 | 1 | 10 |
| *bla*_CTX-M_ | 102 | 5 | 1 | 10 |
